# Supplementary material for: Mechanisms governing the pioneering and redistribution capabilities of the non-classical pioneer PU.1
Source: Nat Commun. 2020 Jan 21;11:402. doi: 10.1038/s41467-019-13960-2 (PMC6972792; doi:10.1038/s41467-019-13960-2)
Supplement: Supplementary file 7 — Source data [file 41467_2019_13960_MOESM7_ESM.zip › Source_Data/FigureS2/FigureS2B_MotifScanOutput/KmeansCluster5/homerResults/motif10.similar.html]

motif10

## Information for motif10

T
C
G
A
A
C
G
T
A
C
T
G
C
T
G
A
A
G
C
T
C
G
A
T
A
C
T
G
C
G
A
T
C
T
G
A
C
G
T
A
T
A
C
G
C
G
A
T
C
A
G
T
  
Reverse Opposite:  

G
T
C
A
C
G
T
A
A
T
G
C
G
A
C
T
A
G
C
T
C
G
T
A
T
G
A
C
G
C
T
A
T
C
G
A
A
G
C
T
A
G
T
C
G
T
C
A
A
G
C
T
  

|  |  |
| --- | --- |
| p-value: | 1e-80 |
| log p-value: | -1.857e+02 |
| Information Content per bp: | 1.743 |
| Number of Target Sequences with motif | 170.0 |
| Percentage of Target Sequences with motif | 4.27% |
| Number of Background Sequences with motif | 203.5 |
| Percentage of Background Sequences with motif | 0.48% |
| Average Position of motif in Targets | 91.4 +/- 66.6bp |
| Average Position of motif in Background | 100.2 +/- 73.2bp |
| Strand Bias (log2 ratio + to - strand density) | -0.6 |
| Multiplicity (# of sites on avg that occur together) | 1.16 |
| Motif File: | file (matrix) reverse opposite |

### Similar de novo motifs found

|  |  |  |  |  |  |  |  |
| --- | --- | --- | --- | --- | --- | --- | --- |
| Rank | Match Score | Redundant Motif | P-value | log P-value | % of Targets | % of Background | Motif file |
| 1 | 0.935 | G C T A C T G A A T G C A G C T G A C T C G T A G T A C C G T A C T G A A G C T A G T C C G T A A G C T C T A G | 1e-80 | -184.459298 | 4.32% | 0.50% | motif file (matrix) |
| 2 | 0.827 | C T G A C G T A C G T A A G T C A C G T A C G T C G T A T A G C C G T A C T G A G A C T A T G C | 1e-70 | -161.969790 | 3.24% | 0.28% | motif file (matrix) |
| 3 | 0.751 | A G C T A G C T A G C T T C A G G C A T T C A G C T G A A G C T C G T A A C T G A C G T T C A G C G T A C T A G | 1e-65 | -150.779859 | 2.54% | 0.14% | motif file (matrix) |
| 4 | 0.862 | T G C A C G T A A G T C A C G T C A G T C G T A A T G C C G T A C T G A G C A T A T G C | 1e-64 | -148.279279 | 3.49% | 0.41% | motif file (matrix) |
| 5 | 0.768 | A C T G C T G A C G A T C G T A A T C G A C G T C T A G C G T A A C T G A C G T A C T G | 1e-58 | -135.098384 | 2.36% | 0.15% | motif file (matrix) |
| 6 | 0.790 | A G T C C G T A A G T C A C G T A G T C G T C A A G T C A C G T C G T A A C G T A T G C C T G A | 1e-48 | -110.569361 | 2.04% | 0.15% | motif file (matrix) |
| 7 | 0.754 | C G T A C G A T G C A T A C T G A G C T T C G A G C T A A C T G G C A T | 1e-45 | -105.455819 | 3.97% | 0.86% | motif file (matrix) |
